# Hydroxy Chalcones and Analogs with Chemopreventive Properties

**DOI:** 10.3390/ijms241310667

**Published:** 2023-06-26

**Authors:** Mihail Lucian Birsa, Laura G. Sarbu

**Affiliations:** Department of Chemistry, Alexandru Ioan Cuza University of Iasi, 11 Carol I Blvd., 700506 Iasi, Romania

**Keywords:** chalcones, antioxidants, anti-inflammatory, chemopreventive

## Abstract

The aim of this review is to highlight the chemopreventive properties of hydroxy-substituted natural and synthetic chalcones along with a number of their analogs. These products display various biological activities, and have many applications against various diseases. Antioxidant and anti-inflammatory properties of chalcones bearing hydroxy substituents are underlined. The influence of hydroxy substituents located on ring A, B, or both are systematized according to the exhibited biological properties.

## 1. Introduction

**Figure 1:**
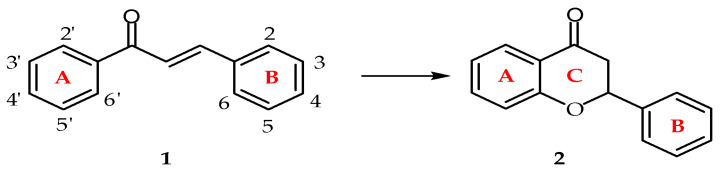
The structure and numbering of chalcones.

Chalcones, chemical description 1,3-diaryl-prop-2-en-1-ones, are important secondary metabolites of plants that belong to the flavonoid family. These metabolites have a general distribution in vegetables and other plants [1,2,3,4]. From the chemical point of view, chalcones present two geometric isomers at the enone moiety, namely the *cis* and *trans* moieties, with the *trans* isomer (**1**)being the most thermodynamically stable isomer (see Figure 1; the numbering used in this paper is described there as well) [5]. Both chalcones, which are considered “open chain” flavonoids (**2**), and their biosynthetic precursors consist of the two aromatic rings depicted in Figure 1 (rings **A** and **B**); the same names and order are used in the description of flavonoids, with the pyrane ring labeled as **C** (see Figure 1).

Associated with the multitude of substitution patterns on the C_6_-C_3_-C_6_ backbone, more than 10,000 flavonoids have been identified to date [6]. This class of compounds has received special attention due to their multiple biological activities. It should be mentioned that natural flavonoids have been used for centuries in traditional medicine for treatment of various diseases, such as for gastrointestinal disease and for wound, urinary, and respiratory infections. The chalcones have not only found favor from the perspectives of synthetic and biosynthetic possibilities; they possess a large spectrum of biological activities as well, including antioxidant, anti-inflammatory, antimicrobial, anticancer, antifungal, and antiviral properties [7,8,9,10,11,12,13,14,15,16,17,18,19,20,21]. 

Bioavailability studies of chalcones and their analogs have indicated possible hindrance and improvement in connection to its pharmaceutical and nutraceutical applications [22]. The mechanisms of action of chalcones have demonstrated the ability to modulate a number of cancer cell lines, as well as to inhibit parasites and other pathological microorganisms. It has been proven that chalcones are involved in the control of a number of signaling molecules and cascades related to disease modification. Clinical studies on chalcones have revealed a lack of adverse effects along with diminished clinical signs and symptoms with decent bioavailability.

Prenylated chalcones and flavonoids have received a special attention in both nutrition and cancer prevention. This is a result of their biological and molecular activities, which have been extensively investigated in vitro and in preclinical studies [23]. These chalcones exhibit antioxidant effects, modulate metabolism of carcinogens by inhibition of distinct phase 1 metabolic enzymes and activation of phase 2 detoxifying enzymes, and display anti-inflammatory properties.

Reactive oxygen species are highly reactive metabolites that are able to induce cellular damage through lipid peroxidation and DNA modification [24]. In this context, compounds possessing antioxidant properties can prevent or reduce oxidative stress, which is involved in diseases such as cancer, diabetes, atherosclerosis, inflammation, and neurodegenerative diseases such as Alzheimer’s disease [25,26]. There have been many reports on chemopreventive compounds that act through antioxidant mechanisms [27,28]. 

In addition to their medicinal applications, chalcones are versatile intermediates in heterocyclic synthesis [29]; in particular, hydroxy-substituted chalcones have several practical applications as color indicators for pH and fluoride ions [30] and for compounds with pH-modulated photoresponsive binding properties [31].

**Scheme 1:**
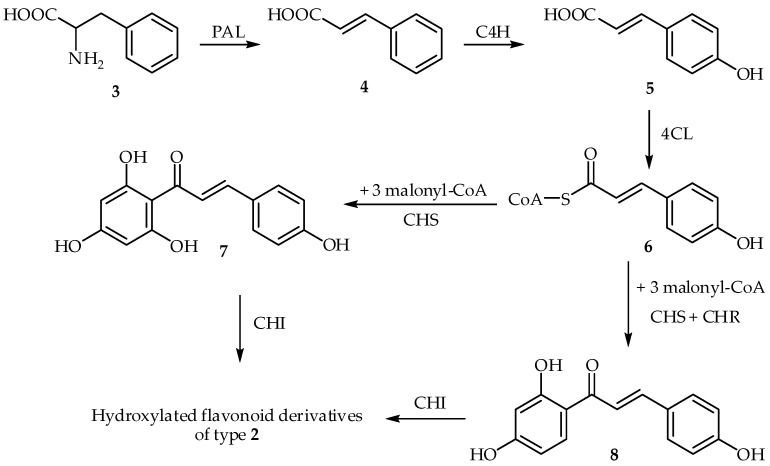
Biosynthesis of chalcones.

As mentioned above, chalcones are precursors in the biosynthesis of benzopyran derivatives and other polyphenolic compounds [32]. Chalcone synthase (CHS) is the vital enzyme in the biosynthesis of chalcones (Scheme 1) [33,34]. Phenylalanine (**3**) is the most important precursor in the biosynthesis of chalcones, and its deamination at the aliphatic chain provides cinnamic acid (**4**). This process is catalyzed by phenylalanine ammonia-lyase (PAL); its hydroxylation at the *para* position of the phenylalanine aromatic ring provides *p*-coumaric acid (**5**) in the presence of cinnamate-4-hydroxylase (C4H) [35]. Succinyl-CoA substitution, catalyzed by 4-coumaroyl-coenzyme A ligase, yields *p*-coumaroyl CoA (**6**). CHS then catalyzes the successive condensation of one molecule of *p*-coumaroyl CoA and three molecules of malonyl-CoA to provide naringerin chalcone (**7**), as depicted in Scheme 1. Formation of the benzopyran ring takes place in the presence of chalcone isomerase (CHI), which leads to the closure of ring **C**, as mentioned above [36]. Two different reaction outputs are operable, differing by the presence or absence of the hydroxyl group at the 6′ position. Using CHS alone, 6′-hydroxychalcones (**7**) (e.g., naringenin–chalcone) is obtained. In the presence of a second enzyme, chalcone reductase (CHR), 6′-deoxychalcone derivatives (**8**) are obtained (Scheme 1) [37].

Many of the natural chalcones bear hydroxyl groups, and most exhibit the biological properties described above. Representative chalcones and dihydrochalcones isolated from natural sources with antioxidant and/or anti-inflammatory properties are summarized in Table 1. The literature contains reports on the synthesis of chalcones [38,39,40]. Generally, synthesis of chalcones is performed via base or acid catalyzed reaction. The synthetic methods and general methodologies for the preparation of synthetic chalcones are presented as follow. Claisen–Schmidt condensation is a simple experimental reaction to obtain chalcones from substituted acetophenone and benzaldehyde derivatives [41]. Grinding [42], microwave [43], and ultrasound techniques [44] have been employed as well. The Witting, Friedel-Crafts acylation, and Julia–Kocienski olefination reactions represent other possible pathways for the synthesis of biologically active chalcones [45]. The Heck [46], Sonogashira [47], and Suzuki–Miyaura [48] reactions are other well-known coupling reactions that can be employed to obtain synthetic chalcones.

This review intends to highlight the antioxidant and anti-inflammatory properties of natural and synthetic hydroxy-substituted chalcones and their analogs.

The Web of Science, Scopus, and SciFinder databases were queried, using “chalcones” as the main keyword and with the search refined using “antioxidants” and “anti-inflammatory” as keywords, with no limit on the date of publication. Therefore, this is a comprehensive review covering all reported articles about hydroxy chalcones with antioxidant and anti-inflammatory properties, starting with the first to be reported. Additional articles have been identified through manual search, including a thorough review of other review articles and relevant references.

## 2. Chemistry and Health Benefits of Hydroxy Chalcones

As mentioned, the chemopreventive role of chalcones is connected with various actions, such as antioxidant and anti-inflammatory properties and the influence of the metabolic enzymes [62]. There are relevant data in the literature about the links between the antioxidant, cytotoxic, and chemopreventive properties of chalcones [63].

An important structural characteristic of chalcones is represented by the hydroxy and methoxy substituents on the A and B rings. These substituents appear to have a main role in the chemoprotective activities of chalcones [64].

An α,β-double bond, a catechol moiety in the B ring, and a free hydroxy substituent at the C3′ position on ring A have been proven to be the structural requirements for DPPH scavenging properties of chalcones [65]. Structure–activity relationship studies on the antioxidant effect of dihydrochalcones indicated that the antioxidant activity of these compounds is dependent on the presence of a hydroxy substituent at the C2′ and C4′ positions. A correlation has been established between peroxynitrite scavenging activity, ionization potential, and lipid peroxidation on the one hand and hydroxy bond dissociation enthalpy on the other [66].

### 2.1. Chalcones with Hydroxy Groups on Ring A

#### 2.1.1. Antioxidant Properties

1-Hydroxynaphthalene-4-trifluoromethylphenyl chalcone (**17**, Table 2) has been synthesized and tested against acetylcholinestarase (49.76 µg/mL) and butyrylcholinesterase (77.82 µg/mL) [67]. Docking studies have indicated the binding of the chalcone to the catalytic active site of acetylcholinesterase. Vanadium complexes of 2′-hydroxychalcone and 1-(1-hydroxy-naphthalen-2-yl)-3-phenyl-propenone have been synthesized and evaluated for their antiradical activity [68,69]. The complex of 2′-hydroxychalcone with vanadium oxychloride proved to be the most active free radical scavengers for DPPH (IC_50_ = 0.03 µg/mL).

The antiproliferative effects of 2′-hydroxy-2,3,4′-trimethoxychalcone (**18**, Table 2) over human hepatoma cells have been investigated [70]. For HepG2 cells, typical nuclear condensation and apoptotic laddering have been observed. The tested chalcone prompted the accumulation of reactive oxygen species in HepG2 cells after 4, 8, and 24 h of treatment. 

The efficacy of the extraction process of biological active components from *Piper methysticum* roots has been reported [71], with pinostrobin chalcone **19** (Table 2) one of the identified extracted phytochemicals. Of a series of solvents, acetone, water, and chloroform have been found, in order, to be the most efficient solvents for the extraction of phenolic and antioxidant compounds from kava roots. Ethanol, methanol, and hexane have been found to be the least effective solvents. 

2-Benzyloxynaphthalene 3′-aminoalkylated-4′-hydroxychalcone **20** (Table 2) has been synthesized and characterized as a potential acetylcholinestarase inhibitor [72]. This compound exhibits important inhibitory activity towards acetylcholinesterase, with an IC_50_ of 1.0 nM. In addition, moderate scavenging properties against DPPH, biometal chelating ability, and better inhibition than donepezil have all been identified. Theoretical studies have revealed that chalcone **20** presents dual binding inhibition of the acetylcholinesterase enzyme.

Protective effects of 4′-hydroxy-3-*m*-tolylchalcone (**21**, Table 2) in indomethacin-induced peptic ulcer in rats has been reported [73]. The same study showed that chalcone increased SOD, PGE2, and GPx activity at a dose-dependent level. A decrease in MDA level was recorded in rats treated with 4′-hydroxy-3-*m*-tolylchalcone as well.

2‘-Hydroxy-2,3,4′,6′-tetramethoxychalcone (**22**, Table 2) has been found to be a potent telomerase inhibitor [74]. This chalcone downregulates the expression of hTERT, and consequently reduces the promotion of hTERT in A549 cells. A reduction in the colony formation ability of A549 cells has been identified upon treatment with 2‘-hydroxy-2,3,4′,6′-tetramethoxychalcone.

#### 2.1.2. Anti-Inflammatory Properties

The methyl ester of 5-cinnamoyl-2-hydroxy-4-methoxybenzoic acid (**23**, Table 2) has been isolated from the methylene chloride fraction of *Dalbergia melanoxylon* [75]. Compared with the lipopolysaccharide model group, compound **23** has been found to reduce the release of nitric oxide in the concentration ranges of 1.2–9.6 and 33.7 μM and to significantly inhibit the secretion of LDH in the ranges of 4.8–9.6 and 16.8–33.7 μM.

2′-Hydroxyhalcones **24**, **25**, and **26** (Table 2) have been found to inhibit iNOS-catalyzed nitric oxide production from lipopolysaccharide-treated RAW 264.7 cells, with IC_50_ values between 7.1–9.6 μM [76]. The most favorable chemical structures have proven to be those with methoxy substituent on the A-ring and a 4-bromine substituent in the B-ring (compounds **25** and **26**). A direct connection has been found between their chemical structures and cellular mechanisms for inhibition of nitric oxide production. 

A chalcone isolated from *Chloranthus henryi*, 2′-hydroxy-4,3′,4′,6′-tetramethoxychalcone (**27**, Table 2), has demonstrated important anti-inflammatory activities in BV2 macrophages [77]. The effects of this chalcone on LPS-induced inflammatory reaction in BV2 microglial cells have been investigated. A correlation was found between the concentration of 2′-hydroxy-4,3′,4′,6′-tetramethoxychalcone and the inhibition of inflammatory enzymes (iNOS, COX-2) and nitric oxide production, as well as the secretion of inflammatory cytokines such as tumor necrosis factor alpha (TNF-α), interleukin(IL)-1β, and IL-6. This same chalcone was able to inhibit ROS species production by downregulating NADPH oxidases. 

2′-Hydroxy-4,4′-dimethoxychalcone (**28**, Table 2) has been isolated from the stems and leaves of *Rhus sylvestris* [78]. It has been demonstrated that this chalcone is able to obstruct inflammatory cytokine secretion in the presence of lipopolysaccharides in the murine RAW264.7 cell line and to inhibit the secretion of TNF-α at very low concentrations (0.01 µM).

4,4′-Dihydroxy-3′-methoxychalcone has been isolated from the stems of *Dracaena usambarensis* [79]. It has been tested for anti-inflammatory properties against GM-CSF, TNF-α, IL-1β, and IL-2 cytokines. At a concentration of 100 μM, this chalcone has been found to be more active than ibuprofen as a standard. 

In the search for developing novel anti-inflammatory compounds, a series of 2′-hydroxy- and 2′,5′-dihydroxychalcones have been synthesized and tested in vitro for their inhibitory effects on the activation of macrophages, microglial cells, neutrophils, and mast cells [80]. Among all hydroxychalcones, 2,2′-dihydroxychalcone has been found to be the most potent inhibitor of the discharge of lysozyme and *β*-glucuronidase from rat neutrophils. 2′,5′-Dialkoxychalcones have shown important inhibitory effects on nitric oxide formation from LPS-stimulated murine microglial cells from the N9 line.

Anti-inflammatory effects on LPS-activated BV-2 microglial cells have been evaluated for a series of hydroxychalcones by rating the production of nitric oxide [81]. Among the tested hydroxychalcones, 2′-hydroxy-3,4,5-trimethoxychalcone (**29**, Table 2) and 2′-hydroxy-3,4,5,3′,4′-pentamethoxychalcone (**30**, Table 2) were established as the most potent compounds, with IC_50_ values of 2.26 and 1.10 µM, respectively. Moreover, 2′-hydroxy-3,4,5,3′,4′-pentamethoxychalcone has been found to reduce iNOS protein expression as well as to downregulate the pro-inflammatory IL-1a, IL-6, and IL-10 cytokines. A structure–activity relationship study has suggested that these strong anti-inflammatory properties are due to the presence of electron-donating hydroxy and methoxy groups on both the A and B rings.

2′-Hydroxy-3,5′,5-trimethoxychalcone (**31**, Table 2, DK-139) has been found to eliminate Toll-like receptor inflammatory replay through inhibition of the Akt/NF-κB pathway in BV2 microglial cells [82]. DK-139 is able to block lipopolysaccharide-induced phosphorylation of IκB and p65/RelA NF-κB, which results in inhibition of the nuclear translocation of NF-κB in BV2 microglial cells. Furthermore, DK-139 diminishes the expression of NF-κB target genes (COX-2, IL-1β, iNOS) in LPS-stimulated BV2 microglial cells. The impact of 2′-hydroxy-3,5′,5-trimethoxychalcone against human lung cancer cells has been investigated as well [83,84].

A study on the relationship between biological activity and the presence of various substituents on the B ring was performed in [85]. It appears that a Michael addition process of chalcone derivatives bearing no hydroxy substituent in the 2′ position is involved in the depletion of cellular glutathione levels.

**Table 2:** Chalcones with hydroxy groups on ring A.

Compd. No.	Common Name	Structure	Biological Activity	Ref
**17**	1-Hydroxynaphthalene-4-trifluoromethylphenyl chalcone	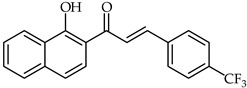	Antioxidant	[67]
**18**	2′-Hydroxy-2,3,4′-trimethoxychalcone	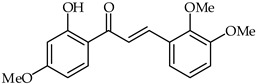	Antioxidant	[70]
**19**	Pinostrobin chalcone	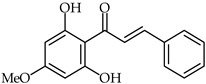	Antioxidant	[71]
**20**	2-Benzyloxynaphthalene 3′-aminoalkylated-4′-hydroxychalcone	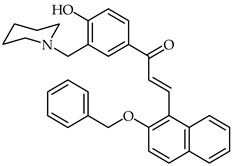	Antioxidant	[72]
**21**	4′-Hydroxy-3-*m*-tolylchalcone	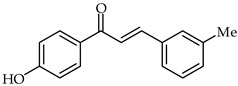	Antioxidant	[73]
**22**	2′-Hydroxy-2,3,4′,6′-tetramethoxychalcone	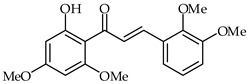	Antioxidant	[74]
**23**	5-Carbomethoxy-4-hydroxy-2-methoxychalcone	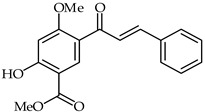	Anti-inflammatory	[75]
**24**	2′-Hydroxy-6′-methoxychalcone	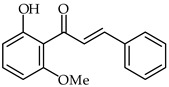	Anti-inflammatory	[76]
**25**	4-Bromo-2′-hydroxy-6′-methoxychalcone	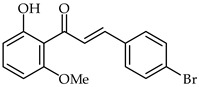	Anti-inflammatory	[76]
**26**	4-Bromo-2′-hydroxy-4′,6′-dimethoxychalcone	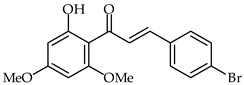	Anti-inflammatory	[76]
**27**	2′-Hydroxy-4,3′,4′,6′-tetramethoxychalcone	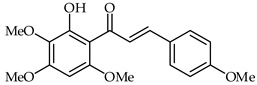	Anti-inflammatory	[77]
**28**	2′-Hydroxy-4,4′-dimethoxychalcone	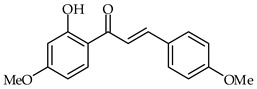	Anti-inflammatory	[78]
**29**	2′-Hydroxy-3,4,5-trimethoxychalcone	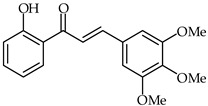	Anti-inflammatory	[81]
**30**	2′-Hydroxy-3,4,5,3′,4′-pentamethoxychalcone	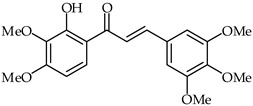	Anti-inflammatory	[81]
**31**	2′-Hydroxy-3,5′,5-trimethoxychalcone	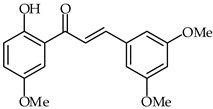	Anti-inflammatory	[82,83,84]
**32**	2′-Hydroxy-4-methoxychalcone	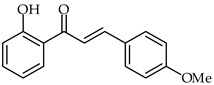	Antioxidant Anti-inflammatory	[86]
**33**	2′-Hydroxy-3′,4′,3,4-tetramethoxychalcone	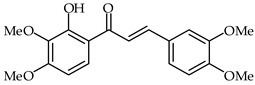	Antioxidant Anti-inflammatory	[87]
**34**	2′-Hydroxy-3,4-dimethoxy-3′,4′-dimethylchalcone	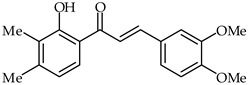	Antioxidant Anti-inflammatory	[87]
**35**	4′-Fluoro-2′-hydroxy-2,3-dimethoxychalcone	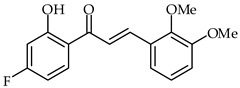	Antioxidant Anti-inflammatory	[88]
**36**	5′-(2-Hydroxycyclohexyl)-6′-hydroxy-2′, 4′,4,6-tetramethoxychalcone	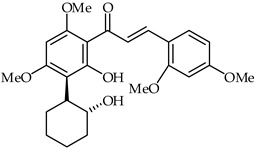	Antioxidant Anti-inflammatory	[89]

#### 2.1.3. Antioxidant and Anti-Inflammatory Properties

It has been recently demonstrated that, in addition to the potential anti-atherosclerosis effects induced by 2′-hydroxy-4-methoxychalcone (**32**, Table 2), it has antioxidant, anti-inflammatory and neuroprotective results as well [86]. 2′-Hydroxy-4-methoxychalcone has been shown to diminish lipopolysaccharide-induced elevations in the levels of oxidative stress and ROS by decreasing gp91phox expression and increasing glutathione (GSH) levels. Moreover, 2′-hydroxy-4-methoxychalcone has been found to attenuate NO, iNOS, and COX-2 levels.

2′-Hydroxy-3′,4′,3,4-tetramethoxychalcone (**33**, Table 2) and 2′-hydroxy-3,4-dimethoxy-3′,4′-dimethylchalcone (**34**, Table 2) have been synthesized from 3,4-dimethoxycinnamic acid and evaluated for their pharmacological properties [87]. These compounds present weak peroxyl scavenging properties and inhibit enzymatic lipid peroxidation, and the former was found to present topical anti-inflammatory effects.

A series of 4′-fluoro-2′-hydroxychalcones have been synthesized and evaluated for their antioxidant, anti-inflammatory, and analgesic properties [88]. Their antioxidant activities were evaluated using the DPPH radical scavenging method (IC_50_ 190 µg/mL) and H_2_O_2_-induced yeast oxidative stress. 4′-Fluoro-2′-hydroxy-2,3-dimethoxychalcone (**35**, Table 2) showed the highest antioxidant activity according to both methods. An in vitro COX assay indicated that 4′-fluoro-2′-hydroxy-4-methoxychalcone has the highest anti-inflammatory activity.

1-[2-Hydroxy-3-(2-hydroxy-cyclohexyl)-4,6-dimethoxy-phenyl]-methanone (**36**) (Table 2) and several synthetic derivatives have been evaluated for their antioxidant and anti-inflammatory activities [89]. DPPH radical scavenging activity and the calculated reducing potential have been used to determine the antioxidant potential. Furthermore, the anti-inflammatory properties of these chalcones have been disclosed using in vitro inhibition assays of β-glucuronidase, trypsin, and diene conjugates. These experimental and theoretical studies indicate that the framework of chalcone **36** represents an important candidate for the design of novel antioxidant and anti-inflammatory agents.

### 2.2. Chalcones with Hydroxy Groups on Ring B

#### 2.2.1. Antioxidant Properties

It has been reported that for chalcones having one or two hydroxyl groups substituted on ring B the antioxidant activity increases in the order 2-OH < 3-OH << 4-OH << 3,4-di-OH [90]. The 3,4-dihydroxy substitution patterns on ring B of chalcones has been found to be the best combination (e.g., 3,4-dihydroxychalcone **37**, Table 3 and 3,4,4′-trihydroxychalcone) for high antioxidant activity [91].

Aminoalkyl-substituted 3,4,4′-trihydroxychalcone chalcones of type **38** (Table 3) have been designed as compounds with antioxidant and anticancer properties [92]. These are related to luteolin as a ring-opened analog. Investigations have revealed that the presence of the aminoethyl moiety in the chalcone structure maintains the antioxidant activity and cytotoxic properties while conferring a benefit in terms of improved pharmacokinetic properties through the possibility of transforming the nitrogen moiety into a water-soluble hydrochloride salt. 

#### 2.2.2. Anti-Inflammatory Properties

4-Hydroxy-4′-methoxychalcone (**39**, Table 3) and 4-hydroxy-3,4′-bis(methoxy)chalcone (**40**, Table 3) have been shown to possess promising anti-inflammatory properties inhibiting TNF-α and IL-6 release [93]. Subsequently, it has been found that acetylated derivatives of these compounds (e.g., 4-acryloyloxy-3,4′-bis(methoxy)chalcone) exhibit important antioxidant properties with respect to H_2_O_2_-induced apoptosis of PC12 Cells (PC12 rat pheochromocytoma) [94].

A structure–relationship activity study of 3-hydroxy-4,3′,4′,5′-tetramethoxychalcone derivatives was realized in order to investigate their anticancer properties and NF-κB inhibitory activity [95]. These compounds showed NF-κB inhibitory activities at low micromolar concentrations.

#### 2.2.3. Antioxidant and Anti-Inflammatory Properties 

A chalcone derivative **41** (Table 3) prepared by acid-catalyzed one-step condensation of 1,3,5-triacetylbenzene with 4-hydroxy-3-methoxybenzaldehyde [96] has been evaluated for free radical DPPH scavenging activity and suppression of lipopolysaccharides-induced nitric oxide generation. Compared with trolox, chalcone **41** has been found to exhibit better influence in terms of DPPH free radical scavenging at a concentration of 10 μM. Moreover, chalcone **41** proved to be a potent suppressor of nitric oxide, with good anti-inflammatory activity at a concentration of 1 µM. 

**Table 3:** Chalcones with hydroxy groups on ring B.

Compd. No.	Common Name	Structure	Biological Activity	Ref
**37**	3,4-Dihydroxychalcone	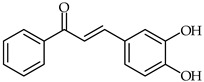	Antioxidant	[91]
**38**	Aminoalkyl-substituted 3,4,4′-trihydroxychalcone chalcones	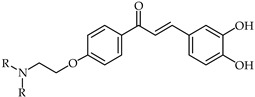	Antioxidant	[92]
**39**	4-Hydroxy-4′-methoxychalcone	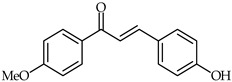	Anti-inflammatory	[93]
**40**	4-Hydroxy-3,4′-dimethoxychalcone	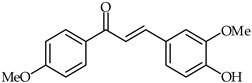	Anti-inflammatory	[93]
**41**	Tris chalcone	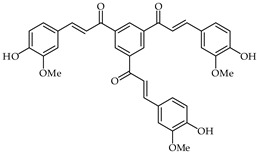	Antioxidant Anti-inflammatory	[96]

### 2.3. Chalcones with Hydroxy Groups on Rings A and B

#### 2.3.1. Antioxidant Properties

2′-Hydroxy chalcone butein (**10**) and dihydroflavone (*S*)-butin have been investigated for their anti-ferroptosis properties [97]. Different antioxidant assays, namely, DPPH, linoleic acid emulsion, and Cu^2+^- and Fe^3+^-reducing antioxidant power assays, have demonstrated that butein **10** exhibits higher antioxidant properties than (*S*)-butin. The conclusions of the aforementioned study revealed that the anti-ferroptotic activities of butein and (S)-butin act as an antioxidant route via the hydrogen atom transfer pathway. This has been explained as being due to the decrease in *π-π* conjugation in butein and the disappearance of the 2′-hydroxy group as a result of biocatalytic isomerization.

A pentahydroxy-substituted chalcone bearing hydroxy groups on the both A and B rings (compound **42**, Table 4) has been found to be an efficient HOCl scavenger [98]. This compound has shown high antioxidant activity, with an IC_50_ value of 1 μM. The neutrophil oxidative inhibition ability has been found to be dependent on the presence of a 2′-hydroxy substituent on the A-ring and other substituents on the B-ring. 

Claisen-Schmidt condensation is a well known method for the synthesis of chalcones. Polysubstituted chalcone **43** (Table 4), containing a 4-fluoro substituent on ring-B, has exhibited good anti-tubercular activity against a strain of *Mycobacterium tuberculosis*, while a related compound with a 2-hydroxy substituent on ring B displayed better antioxidant activity than Gallic acid [99].

Investigations seeking to discover new compounds with antioxidant properties and LOX inhibitory activity have disclosed several 2′-hydroxy-chalcones with diverse substituents on rings A and B [100]. Among these, chalcone **44** (Table 4), with two hydroxy substituents on the ring B, has been identified as possessing a satisfactory LOX inhibition value, with an IC_50_ of 70 μM, good DPPH radical scavenging ability (82.4%), and strong inhibition of lipid peroxidation (82.3%). 

A series of chalcones and their corresponding dihydroderivatives have been synthesized with hydroxy or methoxy substituents in the 2,2′, 3,3′, 4, or 4′ position in order to provide new structures. These structures have shown the ability to inhibit Fe(II)/NADPH-enhanced lipid peroxidation and cytochrome P4501A-dependent 7-cethoxyresorufin-*O*-deethylase (EROD) activity in rat hepatic microsomes [101]. The majority of new synthesized chalcone derivatives have been found to inhibit EROD activity in a dose-dependent manner (0.25–25 μM). Representative compounds from this series include 2′,4,4′-trihydroxychalcone (**45**, Table 4) and 2,4,4′-trihydroxychalcone (**46**, Table 4). These compounds have shown the most promising chemoprotective action against CYP1A activity.

A series of hydroxychalcone derivatives have been prepared to develop potent compounds, showing inhibition of LPO in rat liver microsomes [102]. A representative compound is **47** (Table 4), which bears two hydroxy groups in the *para* positions and two *meta tert*-butyl substituents on the A ring. Moreover, IC_50_ data (9.7 μM) indicate that the presence of isopropyl or *tert*-butyl groups increases the inhibition of LPO.

#### 2.3.2. Anti-Inflammatory Properties

The new chalcone derivatives **48** and **49** (Table 4) have been isolated from *Mallotus philippinensis* [103]. These compounds inhibited NO production and iNOS gene expression in a murine macrophage-like cell line. Moreover, compounds **48** and **49** have been found to downregulate COX-2, IL-1b, and IL-6. 

The important intercellular adhesion molecule-1 (ICAM-1) and vascular cell adhesion molecule-1 (VCAM-1) have been subjected to the action of various anti-inflammatory compounds. 2′,4,4′-Trihydroxychalcone (**50**, Table 4) has been found to decrease the levels of both ICAM-1 and VCAM-1 [104]. An SAR study indicated that the inhibitory activity of these hydroxychalcone derivatives is connected to the presence of a hydroxy group in the 4-position.

### 2.4. Related Chalcones with Hydroxy Groups

#### 2.4.1. Antioxidant Properties

A rapid route to new coumarinyl chalcone has been described in [105]. DPPH radical scavenging indicated chalcone **51** (Table 5) as being the most active compound, with an IC_50_ = 2.07 μM. The preparation of compounds combining coumarin and chalcone has been achieved through the Knoevenagel reaction [106], and their antioxidant properties have been determined through ORAC and ESR assays. The biological activity of these compounds was evaluated against reactive oxygen species, with compound **52** (Table 5) showing the highest ORAC value along with low cytotoxicity and good scavenging capacity, especially against cell death induced by ONOO^–^.

#### 2.4.2. Anti-Inflammatory Properties

The biological activity of four geranyl flavonoid derivatives isolated from *Artocarpus communis* against the human THP-1 monocyte (THP-1) was investigated in [107]. Dihydrochalcone **53** (Table 5) was found to inhibit S100B-induced ROS generation and mRNA expression of IL-6, TNF-α, and COX-2.

A butein (**10**) derivative, 2′,4′,6′-tris(methoxymethoxy)chalcone, has been found to possess important anti-inflammatory activity via the heme oxygenase 1-dependent pathway [108]. As mentioned earlier, the presence of the 2′-hydroxy group has an important role in developing the anti-inflammatory effect. 

A combination of the well known anti-inflammatory compound ibuprofen and an ester with a 4-hydroxy chalcone of type **54** (Table 5) proved to be an important antiproliferative agent towards P450 17A1 prostate cancer cells. This compound was prepared from the reaction of methyl 3-oxo-3*H*-benzocoumarin-2-carboxylate with aryl aldehyde [109].

The synthesis of several 2-benzylidene-1-indanone derivatives and the evaluation of their inhibitory activity on lipopolysaccharides-stimulated reactive oxygen species production in RAW 264.7 macrophages has been reported [110,111]. An SAR study disclosed that the presence of a hydroxy group in the C-5, C-6, or C-7 position of the indanone moiety is important for the inhibition of reactive oxygen species production in LPS-stimulated RAW 264.7 macrophages. A representative compound, 6-hydroxy-2-(2-trifluoromethoxy-benzylidene)-2,3-dihydro-1*H*-inden-1-one (**55**, Table 5), exhibited the strongest inhibitory activity against reactive oxygen species production. 

**Table 5:** Related chalcones with hydroxy groups.

Compd. No.	Common Name	Structure	Biological Activity	Ref
**51**	Chalcone analog	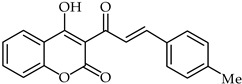	Antioxidant	[105]
**52**	Chalcone analog	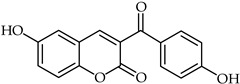	Antioxidant	[106]
**53**	Chalcone analog	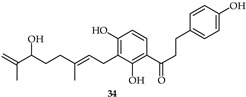	Anti-inflammatory	[106]
**54**	Chalcone analog	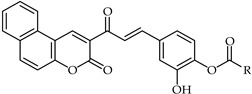	Anti-inflammatory	[109]
**55**	Aurone derivative	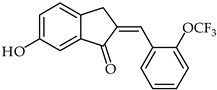	Anti-inflammatory	[110]

## 3. Conclusions

This comprehensive review has highlighted the chemoprotective properties of chalcones bearing hydroxy substituents on rings A and B, covering both their antioxidant and anti-inflammatory properties. The substitution patterns of the two aromatic rings are important in enhancing their biological properties, as evidenced by numerous studies. Several related structures with hydroxy substituents from the broader flavonoid family are mentioned as well. There is no doubt that this class of widely distributed phytochemicals will play an important role in the development of future pharmaceuticals. 

## Figures and Tables

**Table 1:** Representative natural hydroxy chalcones with antioxidant and/or anti-inflammatory properties.

Compd. No.	Common Name	Structure	Biological Activity	Ref
**9**	Isoliquiritigenin	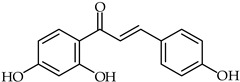	Antioxidant Anti-inflammatory	[49,50,51]
**10**	Butein	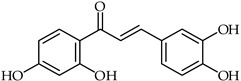	Anti-inflammatory	[52,53]
**11**	Sappanchalcone	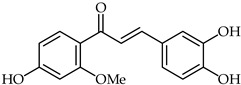	Anti-inflammatory	[54]
**12**	3-Deoxysappanchalcone	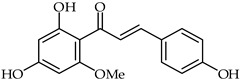	Anti-inflammatory	[55]
**13**	Isobavachalcone	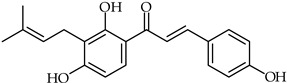	Antioxidant	[56,57]
**14**	Licochalcone	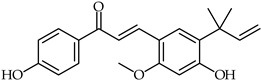	Anti-inflammatory	[58]
**15**	Elastichalcone	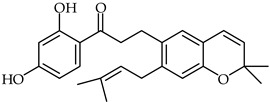	Antioxidant	[59]
**16**	Xanthohumol	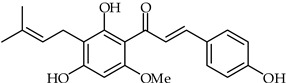	Antioxidant Antiproliferative	[60,61]

**Table 4:** Chalcones with hydroxy groups on rings A and B.

Compd. No.	Common Name	Structure	Biological Activity	Ref
**42**	2′,4′,6′,3,4-Pentahydroxychalcone	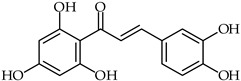	Antioxidant	[98]
**43**	3′-Chloro-4-fluoro-6′-hydroxychalcone	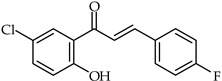	Antioxidant	[99]
**44**	5′-Bromo-3,4,2′,6′-tetrahydroxychalcone	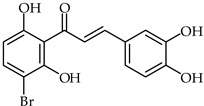	Antioxidant	[100]
**45**	2′,4,4′-Trihydroxychalcone	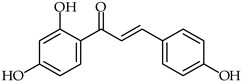	Antioxidant	[101]
**46**	2,4,4′-Trihydroxychalcone	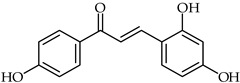	Antioxidant	[101]
**47**	3′,5′-Di-*t*-butyl- 4,4′-dihydroxychalcone	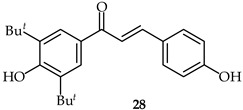	Antioxidant	[102]
**48**	Chalcone from *Mallotus philippinensis*	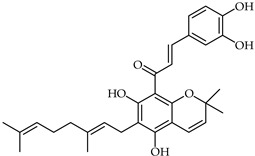	Anti-inflammatory	[103]
**49**	Chalcone from *Mallotus philippinensis*	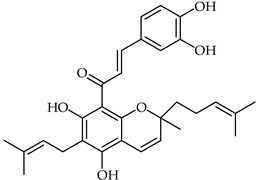	Anti-inflammatory	[103]
**50**	2′,4,4′-Trihydroxychalcone	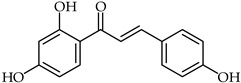	Anti-inflammatory	[104]

## Data Availability

All data used in this study were gathered from open literature sources or scientific journals available under institutional subscription. Data sharing is not applicable to this article.

## Abbreviations

C4H	Cinnamate-4-hydroxylase
CHI	Chalcone isomerase
CHR	Chalcone reductase
CHS	Chalcone synthase
COX	Cyclooxygenase
CoA	Coenzyme A
DPPH	2,2-Diphenyl-1-picrylhydrazyl
HepG2	Liver hepatocellular carcinoma
hTERT	Human telomerase reverse transcriptase
IL	Interleukin
iNOS	Inducible nitric oxide synthase
LDH	Lactate dehydrogenase
LOX	Lipoxygenase
LPO	Lipid peroxidation
LPS	Lipopolysaccharides
MDA	Malondialdehyde
mRNA	Messenger RNA
NADPH	Nicotinamide adenine dinucleotide phosphate
NF-κB	Nuclear factor kappa B
NO	Nitric oxide
PAL	phenylalanine ammonia-lyase
PGE2	Prostaglandin E2
ROS	Reactive oxygen species
SOD	Superoxid dismutase
TNF-α	Tumor necrosis factor alpha
